# *Bombyciellalinzhiensis*, a new species from southern Xizang, China (Lepidoptera, Noctuidae, Noctuinae)

**DOI:** 10.3897/zookeys.1060.71934

**Published:** 2021-09-17

**Authors:** Enyong Chen, Zhaohui Pan, Anton V. Volynkin, Aidas Saldaitis, Balazs Benedek

**Affiliations:** 1 Key Laboratory of Forest Ecology in Tibet Plateau (Institute of Plateau Ecology, Tibet Agricultural and Animal Husbandry University), Ministry of Education, Linzhi 860000, China Tibet Agricultural and Animal Husbandry University Linzhi China; 2 Altai State University, Lenina Avenue, 61, RF-656049, Barnaul, Russia Altai State University Barnaul Russia; 3 Nature Research Centre, Akademijos str., 2, LT-08412, Vilnius-21, Lithuania Nature Research Centre Vilnius Lithuania; 4 H-2045 Törökbálint, Árpád u. 53, Hungary Unaffiliated Törökbálint Hungary

**Keywords:** Male genitalia, morphology, Owlet moth, taxonomy, Xylenina, Xylenini

## Abstract

A new species of the genus *Bombyciella* Draudt, 1950, *Bombyciellalinzhiensis***sp. nov.**, is described from the Linzhi (Nyingchi) Prefecture in southern Xizang (China), following a diagnostic comparison with *B.talpa* Draudt, 1950 and *B.antra* Saldaitis, Benedek, Behounek & Stüning, 2014. The adults and the male genitalia of the new and related species are illustrated.

## Introduction

*Bombyciella* Draudt, 1950 is a noctuid genus distributed in mountain areas in south-western and northern China. The genus is closely related to the Holarctic *Brachylomia* Hampson, 1906 (Saldaitis et al. 2014) and belongs to the subtribe Xylenina of the tribe Xylenini of the subfamily Noctuinae (Lafontaine and Schmidt 2010; Zahiri et al. 2011). The genus has recently been revised by Saldaitis et al. (2014) and currently comprises two valid species. The male genitalia structures of *Bombyciella* and *Brachylomia* are very similar and display no distinctive apomorphic features. Therefore, it is possible that these taxa represent one genus, an issue that can be resolved in the future by using molecular methods through a multi-gene phylogenetic analysis covering these and other related genera.

During an entomological survey in the southern Xizang Province of China, a long series of unusual dark-coloured *Bombyciella* specimens was collected by the senior and the second authors. After comparing the male genitalia structures of these specimens with the other two species in the genus, they proved to be diagnostic and the specimens are therefore considered to represent a new species which is here described.

## Materials and methods

Abbreviations for private and institutional collections used herein are as follows:

**AFM** collection of Alessandro Floriani (Milan, Italy);

**TAAHU** Tibet Agricultural and Animal Husbandry University (Linzhi, China);

**WIGJ** World Insect Gallery (Joniškis, Lithuania);

**ZFMK**Zoological Research Museum Alexander Koenig (Zoologisches Forschungsmuseum Alexander Koenig, Bonn, Germany);

**ZSM**Bavarian State Collection of Zoology (Zoologische Staatssammlung München, Munich, Germany).

Other abbreviations used in the illustrations are:

**HT** holotype;

**LT** lectotype;

**PT** paratype.

The male genitalia terminology follows Fibiger (1997) and Kononenko (2010).

## Results

### Noctuidae Latreille, 1809

**Noctuinae Latreille, 1809**

**Xylenini Guenée, 1837**

#### Xylenina Guenée, 1837

##### Bombyciella linzhiensis sp. nov.

Taxon classificationAnimaliaLepidopteraNoctuidae

7BDD9D5C-F8BF-51FD-8E09-1CBBB2B98200

http://zoobank.org/08CFBD59-8B98-40C5-91B3-B0EAF8E96C2B

Figs 1–5 , 9 , 10

###### Type material.

**Figures 1–8.**
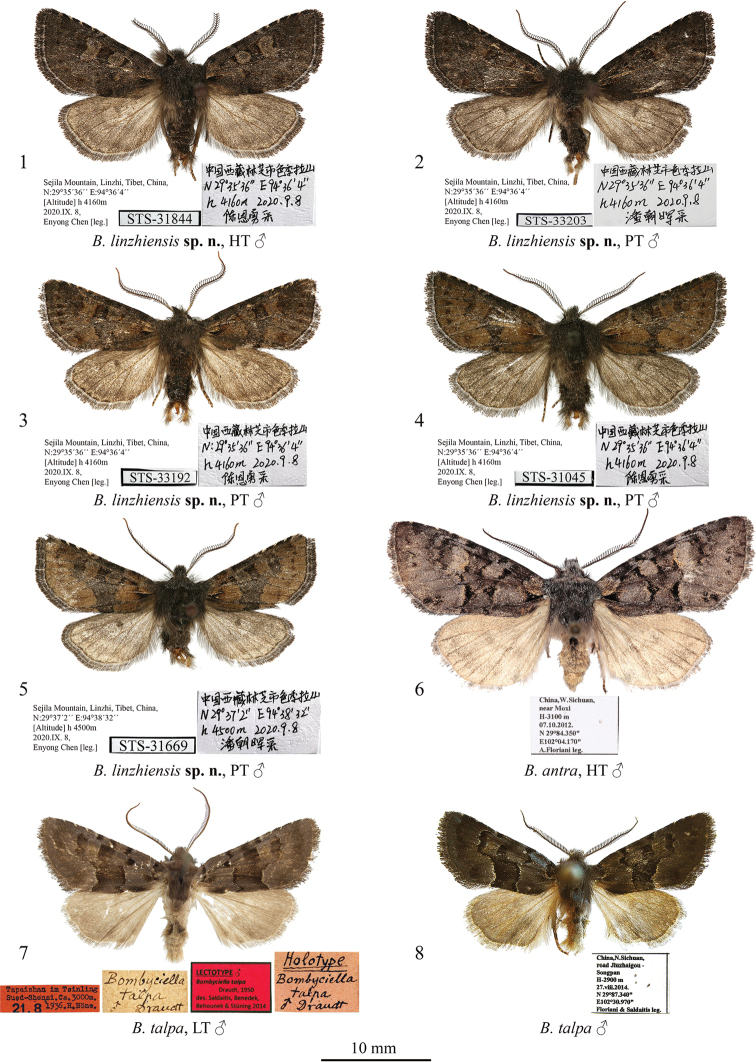
*Bombyciella* spp., adults. Depositories of the specimens **1–5** in TAAHU**6** in ZSM**7** in ZFMK (photo by D. Stüning) **8** in AFM.

***Holotype*:** male (Fig. 1), “Sejila Mountain, Linzhi, Tibet, China, 29°35'36"N, 94°36'4"E, 4160 m a.s.l., 8.IX.2020, Enyong Chen [leg.]” (in Chinese), unique number: STS-31844, gen. prep. in glycerol by Enyong Chen (coll. TAAHU). ***Paratypes***: 31 males (Figs 2–5), with the same data as in the holotype, unique numbers: STS-31032, STS-31044, STS-31045, STS-31046, STS-31047, STS-31048, STS-31049, STS-31050, STS-31051, STS-31052, STS-31101, STS-31102, STS-31127, STS-31129, STS-31131, STS-31133, STS-31136, STS-31137, STS-31140, STS-31840, STS-31845, STS-31851, STS-33192, STS-33202, STS-33203, STS-33419, STS-33421, STS-33424, STS-33427, STS-33433, STS-33438, gen. prep. in glycerol by Enyong Chen; 6 males, “Sejila Mountain, Linzhi, Tibet, China, 29°37'2"N, 94°38'32"E, 4500 m a.s.l., 8.IX.2020, Zhaohui Pan [leg.]” (in Chinese), unique numbers: STS-29906, STS-29907, STS-29908, STS-31666, STS-31668, STS-31669, gen. preps. in glycerol by Enyong Chen (colls TAAHU and WIGJ).

###### Diagnosis.

**Figures 9–12.**
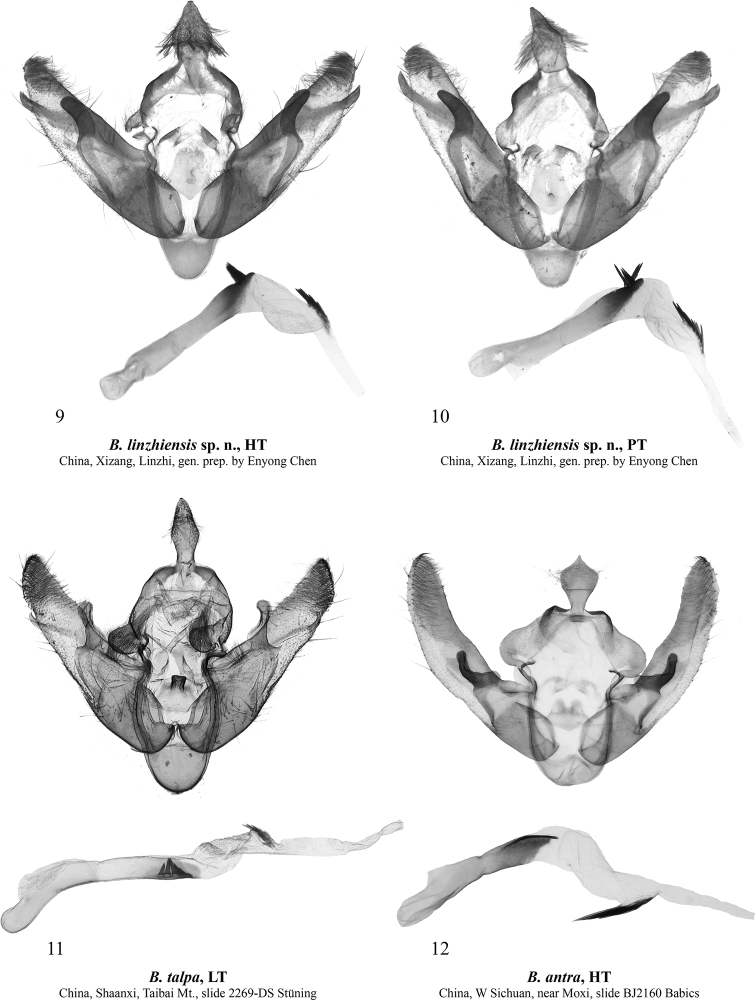
*Bombyciella* spp., male genitalia. Depositories of the specimens dissected **9, 10** in TAAHU**11** in ZFMK (photo by D. Stüning) **12** in AFM.

The new species (Figs 1–5) is externally similar to the type species of the genus *Bombyciella*, namely *Bombyciellatalpa* Draudt, 1950 (Figs 7, 8), but it can be distinguished by its darker body colouration, the more tapered forewing apex, the more indistinct transverse lines of the forewing and the dark grey hindwing having a distinct discal spot, whereas in *B.talpa* the hindwing is pale brown and the discal spot is absent or faint. *Bombyciellalinzhiensis* sp. nov. differs clearly from another congener, *B.antra* Saldaitis, Benedek, Behounek & Stüning, 2014 (Fig. 6), by its significantly smaller size (the forewing length is 26 mm vs 39 mm in *B.antra*), the blackish brown head and thorax (bluish grey in *B.antra*), the blackish brown abdomen (pale brown in *B.antra*), the straight costal margin (slightly convex in *B.antra*), the more convex outer margin of the forewing, the blackish brown or olive brown forewing colouration (it is bluish grey in *B.antra*), and the dark grey hindwing with a distinct discal spot (whereas in *B.antra* the hindwing is pale brown with a grey suffusion and lacks a discal spot but has a diffuse thin transverse line). The male genital capsule of *B.linzhiensis* sp. nov. (Figs 9, 10) is reminiscent of that of *B.talpa* (Fig. 11) but differs by the wider uncus, the considerably smaller, tubercle-like medio-ventral process of juxta, the more apically rounded valva, the downward pointing and evenly-wide clasper tapered only apically (it is subapically constricted with an upward pointing apex in *B.talpa*), and the thicker and longer costal process. Additionally, the peniculus of the new species is shorter than in *B.talpa* and the saccus is somewhat shorter and narrower. The aedeagus of *B.linzhiensis* sp. nov. is shorter (in relation to the tegumen-vinculum complex) and less sub-proximally curved than in *B.talpa*. The carina of the new species bears a bunch of spines directed ventrally whereas that of *B.talpa* bears a row of laterally directed spikes. The vesica of *B.linzhiensis* sp. nov. is broader than that of *B.talpa* and bears distally a cluster of more robust spike-like cornuti. Compared to that of *B.antra* (Fig. 12), the male genital capsule of *B.linzhiensis* sp. nov. has a more distally elongated and apically obtuse uncus (apically pointed in *B.antra*), a basally wider and less elongated valva having a straight dorsal margin and a well-developed costal process protruding beyond the ventral margin, whereas in *B.antra* the dorsal margin of valva is medially slightly convex and distally somewhat curved dorsally, and the costal process is reduced. The clasper of the new species is thicker and evenly down-curved whereas it is up-curved in *B.antra*. Additionally, the peniculus of the new species is shorter and narrower than that of *B.antra*, the juxta is markedly longer and the saccus is narrower and somewhat longer. In the aedeagus of *B.linzhiensis* sp. nov., the carina bears a bunch of spines whereas it is plate-like without spines in *B.antra*. The vesica of the new species is somewhat shorter and broader (in proportion to the aedeagus size) than in *B.antra* and bears a cluster of markedly shorter cornuti distally.

###### Description.

**Male.** Forewing length 26–27 mm (holotype: 26 mm). Antenna shortly bipectinate. Head and thorax blackish grey with suffusion of pale grey scales. Forewing triangular with tapered apex and almost straight costa and convex termen. Forewing ground colour varies from blackish brown to olive brown. Subbasal dash short, black. Antemedial line irregularly sinuous, black, indistinct, oblique outwards posteriorly. Postmedial line indistinct, dentate on veins, loop-like curved anteriorly and oblique inwards posteriorly. In olive brown form, medial area intensely suffused with black scales. Orbicular stigma elliptical, from greyish- to olive brown. Reniform stigma wide, slightly dilated anteriorly, from greyish- to olive brown. Subterminal line evenly curved, parallel to termen, brown, interrupted into diffuse spots on veins. Terminal line blackish brown, thin. Costal margin intensely suffused with blackish scales, with three thin and short whitish subapical dashes. Cilia from dark grey to blackish grey. Hindwing dark grey with brown suffusion on veins, thin brown terminal line and comma-like, dark brownish grey discal spot. Hindwing cilia brownish grey. Abdomen blackish brown, with suffusion of pale grey scales along segment edges.

***Male genitalia*** (Figs 9, 10). Tegumen short, with arcuate arms. Penicular lobe small, rounded. Vinculum ca 1.6 times longer than tegumen, with more or less U-shaped saccus. Valva elongated with almost parallel margins medially, distally tapered and apically rounded, with densely setose apex. Sacculus short (ca 1/3 of valva length) and broad (ca 4/5 of valva width). Clasper flattened, smoothly downward pointing, distally tapered and apically rounded. Costal process (= digitus sensu Forbes nec Pierce) elongated, blade-like, apically pointed, directed ventro-distally and protruding beyond the ventral edge of valva. Uncus arrowhead-shaped, dorso-ventrally flattened, apically obtuse, densely setose. Tuba analis narrow and membranous. Juxta pentagonal shield-like, with a small tubercle-like medio-ventral process. Aedeagus elongated and narrow (length to width ratio ca 7.5:1), somewhat dilated proximally and distally, with short (less than 1/5 of aedeagus length) and rounded coecum. Carina triangular plate-like, apically rounded, bearing bunch-like cluster of 6–8 ventrally directed, thin spikes of various lengths. Vesica relatively short (ca 1/2 of aedeagus length), dorsally projected, slightly medially broader than aedeagus, somewhat twisted subbasally and dilated distally with a stripe of few robust spine-like cornuti before the gonopore.

**Female.** Unknown.

###### Distribution.

**Figure 13.**
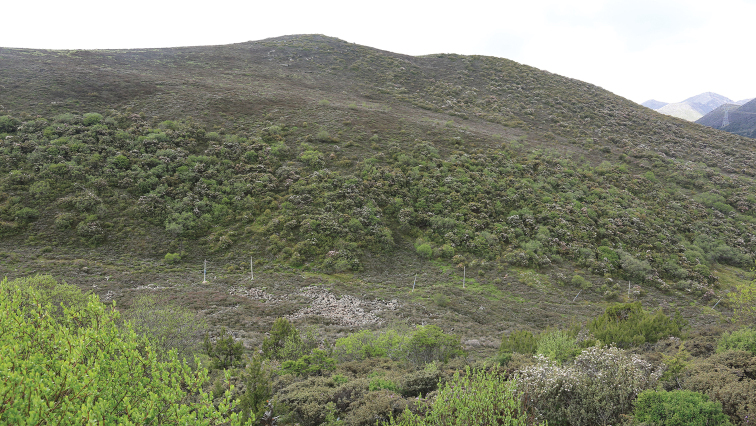
South of the collecting locality on Sejila Mountain, Linzhi, Tibet, China, 29°35'36"N, 94°36'4"E, 4160 m a.s.l., the type locality of *Bombyciellalinzhiensis* (photo by Enyong Chen).

**Figure 14.**
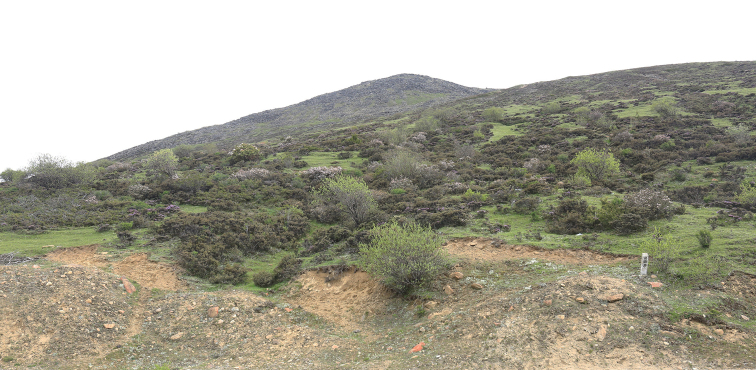
North of the collecting locality on Sejila Mountain, Linzhi, Tibet, China, 29°35'36"N, 94°36'4"E, 4160 m a.s.l., the type locality of *Bombyciellalinzhiensis* (photo by Enyong Chen).

The new species is known only from Sejila Mountain in southern Xizang Province of China (Figs 13, 14).

###### Etymology.

The specific epithet refers to the type locality located in the Linzhi (Nyingchi) prefecture of Xizang.

## Supplementary Material

XML Treatment for Bombyciella linzhiensis
